# Effects of Sulfur Assimilation in *Pseudomonas fluorescens* SS101 on Growth, Defense, and Metabolome of Different *Brassicaceae*

**DOI:** 10.3390/biom11111704

**Published:** 2021-11-16

**Authors:** Je-Seung Jeon, Desalegn W. Etalo, Natalia Carreno-Quintero, Ric C. H. de Vos, Jos M. Raaijmakers

**Affiliations:** 1 Department of Microbial Ecology, Netherlands Institute of Ecology, 6708 PB Wageningen, The Netherlands; jsjeoncy@gmail.com (J.-S.J.); D.Etalo@nioo.knaw.nl (D.W.E.); 2 Institute of Biology, Leiden University, 2333 BE Leiden, The Netherlands; 3 KeyGene, Agro Business Park 90, 6708 PW Wageningen, The Netherlands; natalia.carreno-quintero@keygene.com; 4 Business Unit Bioscience, Wageningen Plant Research, Wageningen University and Research (Wageningen-UR), 6700 AA Wageningen, The Netherlands; ric.devos@wur.nl

**Keywords:** flavonoids, glucosinolates (GLSs), induced systemic resistance, *Pseudomonas fluorescens*, plant growth promotion, plant metabolomics

## Abstract

Genome-wide analysis of plant-growth-promoting *Pseudomonas fluorescens* strain SS101 (*Pf*SS101) followed by site-directed mutagenesis previously suggested that sulfur assimilation may play an important role in growth promotion and induced systemic resistance in *Arabidopsis*. Here, we investigated the effects of sulfur metabolism in *Pf*SS101 on growth, defense, and shoot metabolomes of *Arabidopsis* and the Brassica crop, Broccoli. Root tips of seedlings of *Arabidopsis* and two Broccoli cultivars were treated with *Pf*SS101 or with a mutant disrupted in the adenylsulfate reductase *cysH*, a key gene in cysteine and methionine biosynthesis. Phenotyping of plants treated with wild-type *Pf*SS101 or its *cysH* mutant revealed that sulfur assimilation in *Pf*SS101 was associated with enhanced growth of *Arabidopsis* but with a reduction in shoot biomass of two Broccoli cultivars. Untargeted metabolomics revealed that *cysH*-mediated sulfur assimilation in *Pf*SS101 had significant effects on shoot chemistry of *Arabidopsis*, in particular on chain elongation of aliphatic glucosinolates (GLSs) and on indole metabolites, including camalexin and the growth hormone indole-3-acetic acid. In Broccoli, *Pf*SS101 sulfur assimilation significantly upregulated the relative abundance of several shoot metabolites, in particular, indolic GLSs and phenylpropanoids. These metabolome changes in Broccoli plants coincided with *Pf*SS101-mediated suppression of leaf infections by *Xanthomonas campestris*. Our study showed the metabolic interconnectedness of plants and their root-associated microbiota.

## 1. Introduction

The genus *Pseudomonas* is an abundant member of the plant microbiome, particularly of the rhizosphere. Various studies have shown that different strains of root-associated *Pseudomonas* species can promote plant growth, alter root architecture, and induce systemic resistance [1,2,3]. By screening a genome-wide library of approximately 7500 random transposon mutants, we previously identified specific genes in root-colonizing *Pseudomonas fluorescens* strain SS101 (*Pf*SS101) that were associated with growth promotion and induced systemic resistance in *Arabidopsis* [4]. Twenty-one mutants were identified with a compromised ability to promote plant growth, to alter root architecture, or to trigger systemic resistance against the bacterial leaf pathogen *Pseudomonas syringae* pv. tomato (Pst). Subsequent validation by site-directed mutagenesis and genetic complementation of specific mutants in *Pf*SS101demonstrated that the phosphogluconate dehydratase gene *edd*, the response regulator gene *colR,* and the adenylsulfate reductase gene *cysH* play important roles in plant growth promotion, alteration of root architecture, and induced systemic resistance (ISR). *cysH* is a key gene involved in sulfur assimilation and in the biosynthesis of the amino acids cysteine and methionine. Transcriptome analysis of *Arabidopsis* further revealed that biosynthetic processes associated with sulfur compounds (in particular cysteine and GLSs) were the most significantly enriched in seedlings treated with *Pf*SS101 when compared to control plants and to plants treated with the *cysH* mutant [4]. These results suggested that *Pf*SS101 modulates sulfur metabolism in *Arabidopsis*, confirming and extending earlier results from Meldau et al. [5] and Aziz et al. [6] who attributed modulation of sulfur metabolism as a key mechanism of growth promotion and induction of lateral roots of tobacco and *Arabidopsis* by different *Bacillus* strains.

In plants, sulfate (SO_4_^2−^) taken up by roots from the environment is turned into adenosine 5′-phosphosulfate (APS) and subsequently reduced to sulfite (SO_3_^2−^) and then to sulfide (S^2−^). The condensation of S^2−^ and an amino acid derivative O-acetylserine results in cysteine. In plants, this key amino acid is incorporated into proteins and glutathione or serves as the sulfur donor of methionine and sulfur-containing secondary metabolites [7]. Previously, we demonstrated that plant-growth-promoting *Pseudomonas fluorescens* strain SS101 (*Pf*SS101) increased the levels of glucosinolates (GLSs), coumarins, flavonoids, and camalexin in shoots and roots of *Arabidopsis* [8]. In a recent study, Kopriva et al. [9] elegantly showed, via genome-wide-association analysis and bioassays with *Arabidopsis* mutants, that the sulfur-containing indolic compound camalexin is associated with the growth-promoting activity of *Pseudomonas* sp. CH267. In summary, the above-mentioned studies indicate that rhizobacteria can influence their host metabolism. However, if and to what extent a particular bacterial trait influences host metabolism, growth, and tolerance to biotic stresses remains largely elusive.

The aim of the present study was to investigate the effects of sulfur metabolism (i.e., the effects of the *cysH* mutation) in *Pf* SS101 on the shoot metabolome of *Arabidopsis*. To that end, we adopted a non-targeted comparative metabolomics approach to assess the differences between the shoot metabolomes of *Arabidopsis* seedlings treated with either *Pf*SS101 or *cysH*-mutant 20H12. Furthermore, we investigated if strain *Pf*SS101 can induce similar phenotypic and metabolome changes in shoots of the related Brassicaceous crop plant Broccoli, *Brassica oleracea* var. *italica*, and if these changes are associated with sulfur metabolism. For Broccoli, two cultivars were selected based on their contrasting levels of GLSs (glucoiberin, glucoraphanin, and glucobrassicin) and thereby possible differences in sulfur needs. The *Pf*SS101-mediated phenotypic responses investigated for the Broccoli cultivars included root and shoot growth as well as induced systemic resistance against two pathovars of *Xanthomonas campestris*, an important bacterial leaf pathogen of Broccoli and other cruciferous crops [10].

## 2. Materials and Methods

### 2.1. Plant Material and Growth

Seeds of *Arabidopsis thaliana* ecotype Columbia (Col-0) were surface-sterilized as previously described [8]. Seeds of two Broccoli (*Brassica oleracea* var. *italica*) cultivars, namely Coronado and Malibu, were kindly provided by Bejo Seeds (Trambaan1, 1749 CZ Warmenhuizen, The Netherlands). Cultivar Coronado has higher levels of specific GLSs (glucoiberin, glucoraphanin, and glucobrassicin) than cultivar Malibu. Surface sterilization of the Broccoli seeds was performed by immersing 2–3 g of seeds for 30 min in 30 mL of 1% (*v*/*v*) sodium hypochlorite supplemented with 0.1% (*v*/*v*) of Tween 20 followed by three washes with ample sterile distilled water. For the *Arabidopsis* assays, sterile seeds were sown on 90-mm-diameter Petri dishes containing 20 mL half-strength Murashige and Skoog (MS) agar medium, containing 0.5% sucrose (*w*/*v*) and 1.2% plant agar (*w*/*v*). For the Broccoli assays, five sterile seeds were sown on 140-mm-diameter petri dishes containing 50 mL of half-strength MS agar. The plates were then placed in a climate chamber maintained at 21 °C/21 °C day/night; 180 µmol light m^−2^s^−1^, 16 h light/8 h dark cycle, and 70% relative humidity. After a week of *Arabidopsis* growth and five days of Broccoli growth, roots of each seedling were inoculated with a 2 µL of bacterial suspension (~10^9^ cells mL^−1^). After inoculation, plants were placed back in the same growth chamber until harvest.

### 2.2. Bacterial Strains and Culture Conditions

*Pseudomonas fluorescens* strain SS101 (*Pf*SS101 (Taxonomy ID: 1038924)) was originally isolated from the wheat rhizosphere [11]. *cysH* (adenylylsulfate reductase)-mutant 20H12 of *Pf*SS101 was generated by site-directed mutagenesis as described by Cheng et al. [4]. *Pf*SS101 and mutant 20H12 were cultured in King’s medium B (KB) at 25 °C for 16 h. Broccoli leaf pathogens *Xanthomonas campestris* pv. armoraciae (*Xca*) and *Xanthomonas campestris* pv. campestris (*Xcc*) were kindly provided by Bejo Seeds and were cultured in Luria Bertani (LB) medium (Lennox, Carl Roth GmbH, Germany) at 25 °C for 16 h. Bacterial cells were collected by centrifugation, washed three times and resuspended in with 10 mM MgSO_4_ to a final density of 10^9^ cells mL^−1^ (OD600 = 1.0).

### 2.3. Bioassay for Induced Resistance against the Leaf Pathogen Xanthomonas 

The roots of the Broccoli plants were treated with *Pf*SS101 or the *cysH* mutant and grown for 11 days. The first true leaves of the primed plants were then pierced and 2 µL (10^9^ cells mL^−1^) of inoculum of the bacterial pathogens *Xca* and *Xcc* was applied. Ten days after pathogen challenge, a 0–5 ordinal scale that scores the migration of the lesion from the inoculation spot to other parts of the shoot was used (Figure S1). Scale: 1 = no necrosis or migration, 2 = necrosis of the treated leaf, 3 = migration of the lesion to the leafstalk of the treated leaf, 4 = visible necrotic or water-soaked lesions of the neighboring (non-treated) leaf, and 5 = infection of the entire shoot. Severity values were converted to a 0 to 100 disease severity index (DSI) according to following equation used by [12]: DSI (%) = ∑ (scores of all plants)/(maximum disease score × (total number of plants)) × 100. Next to this disease severity assessment, treated Broccoli shoots were collected, ground in a sterile mortar and suspended in 50 mL Falcon tube to measure their biomass. Samples were then vortexed for 60 s in 10 mM MgSO_4_, sonicated for 60 s, and again vortexed for 15 s. The suspension was plated in serial dilution onto King medium B agar plates containing 100 µg mL^−1^ delvocid (DSM) to inhibit fungal growth and incubated for 3 days at 25 °C. Colonies with the typical phenotypes of *Xca* and *Xcc* were counted and expressed as colony-forming units (cfu) mg^−1^ of shoot fresh weight.

### 2.4. Plant Metabolite Analysis

#### 2.4.1. Sample Preparation

Shoots of *Arabidopsis* and Broccoli were harvested at 11 days after root treatment with buffer (control), *Pf*SS101, or mutant 20H12. For each plant species/cultivar X rhizobacteria combination, four biological replicates were used with 10 *Arabidopsis* and five Broccoli seedlings per replicate. In brief, shoots were snap frozen in liquid nitrogen and ground to fine powder under continuous cooling and kept at −80 °C until further use. To extract semi-polar secondary metabolites, 300 µL of 99.89% methanol containing 0.13% (*v*/*v*) formic acid was added to 100 mg plant material in 2 mL round bottom Eppendorf tubes, mixed, and sonicated for 15 min followed by centrifugation for 15 min at 20,000× *g*. The supernatants were transferred to 96-well filter plates (AcroPrepTM, 350 µL, 0.45µm, PALL, Life Sciences, Crailsheim, Germany), vacuum filtrated to the 96-deep-well autosampler plates (Waters, Milford, CT, USA) using a Genesis Workstation (Tecan Systems, Crailsheim, Germany).

#### 2.4.2. Metabolite Analysis

An UltiMate 3000 U-HPLC system (Dionex, Idstein, Germany) was employed to create a 45-min linear gradient of 5–35% (*v*/*v*) acetonitrile in 0.1% (*v*/*v*) formic acid (FA) in water at a flow rate of 0.19 mL per min. Then, 5 µL of each extract was injected and compounds were separated on a Luna C18 column (2.0 × 150 mm, 3 µm; Phenomenex, Torrance, CA, USA) maintained at 40 °C [13]. The detection of compounds eluting from the column was carried out with a Q-Exactive Plus Orbitrap FTMS mass spectrometer (Thermo Scientific, Waltham, MA, USA). Full scan MS data were generated with electrospray in switching positive/negative ionization mode at a mass resolution of 35,000 (FWHM at *m*/*z* 200) in a range of *m*/*z* 95–1350. Subsequent MS/MS experiments for identification of selected metabolites were performed with separate positive or negative electrospray ionization at a normalized collision energy of 27 and a mass resolution of 17,500. The ionization voltage was optimized at 3.5 kV for positive mode and 2.5 kV for negative mode; capillary temperature was set at 250 °C; the auxiliary gas heater temperature was set to 220 °C; and sheath gas, auxiliary gas and the sweep gas flow were optimized at 36, 10, and 1 arbitrary units, respectively. Automatic gain control was set a 3e^6^ and the injection time at 100 ms. External mass calibration with formic acid clusters was performed in both positive and negative ionization modes before each sample series.

#### 2.4.3. Non-Targeted LC–MS Data Processing and Analysis

Baseline-corrected mass features of the raw LC–MS data were extracted and aligned using Metalign software 3.0. [14]. The mass features were considered as a reproducible signal if they were detected in at least three biological replicates of a treatment with a signal intensity three times higher than the background noise value calculated by Metalign. Then, mass features originating from the same metabolite, as generated in the ion source, were clustered based on similarities in both retention time and relative abundance across all measured samples, using MSClust software [12]. This software removes metabolite signal redundancy and generates so-called centrotypes, representing reconstructed putative metabolites with their in-source mass spectra. The relative abundance of each compound in a given sample is represented by the measured ion counts (MIC), which is the sum of the ion count values (corrected by their centrotype membership) for all clustered ions. ANOVA with Benjamini–Hochberg false discovery rate (BH-FDR) correction (*p* < 0.05) and fold changes more or less than 2.0, either increased or decreased, were applied to identify mass signals that were significantly changed in bacteria-treated compared to control samples. Then, we performed a bootstrapping analysis to remove duplicate metabolites between the two ionization modes (positive and negative) incorporating retention time and mass relationship between two metabolites. Data transformation and scaling were performed in GeneMaths XT (version 1.6.1, Applied Maths, Belgium). Transformed and scaled values were used for hierarchical cluster analysis using Pearson’s correlation coefficient and the unweighted pair group method with arithmetic mean (UPGMA). 

Annotation of metabolite signals was carried out based on manual selection of the pseudomolecular ions from the masses in the MSClust-reconstructed metabolites, followed by matching the observed accurate masses plus retention times to metabolites previously detected and reported in *Arabidopsis* and Broccoli using a similar LC–MS system and identical chromatographic conditions (e.g., van de Mortel et al. [8]). If molecular ion masses were not yet present in this experimentally obtained dedicated metabolite database, KEGG and HMDB MS/MS databases mounted on the MAGMa online tool [12] were primarily applied, allowing a maximum deviation of observed mass from calculated mass of 5 ppm. In addition, some annotations were complemented by the aid of other publicly available compound libraries, including PubChem [15] and Metlin [16].

### 2.5. Statistical Analysis

Changes in shoot biomass and pathogen incidence between treatments were analyzed with R Studio software (Version 3.6.1) following similar methodology used in our previous studies [4,8,17]. The data for plant shoot and root biomass were statistically analyzed by two-way analysis of variance (ANOVA). A Tukey HSD test was used to separate group mean values when the ANOVA was significant at *p* < 0.05. For the disease severity data, beta regression analysis was employed to examine the interaction effect of two independent variables (Rhizobacteria and Broccoli cultivars) on disease severity of the two bacterial pathogens using (“betareg”) package in R [18]. The correlation between bacterial root colonization and percent change in biomass was also calculated in R (Figure S3).

## 3. Results

### 3.1. Role of the cysH Gene of P. fluorescens SS101 in Plant Growth Promotion

**Figure 1:**
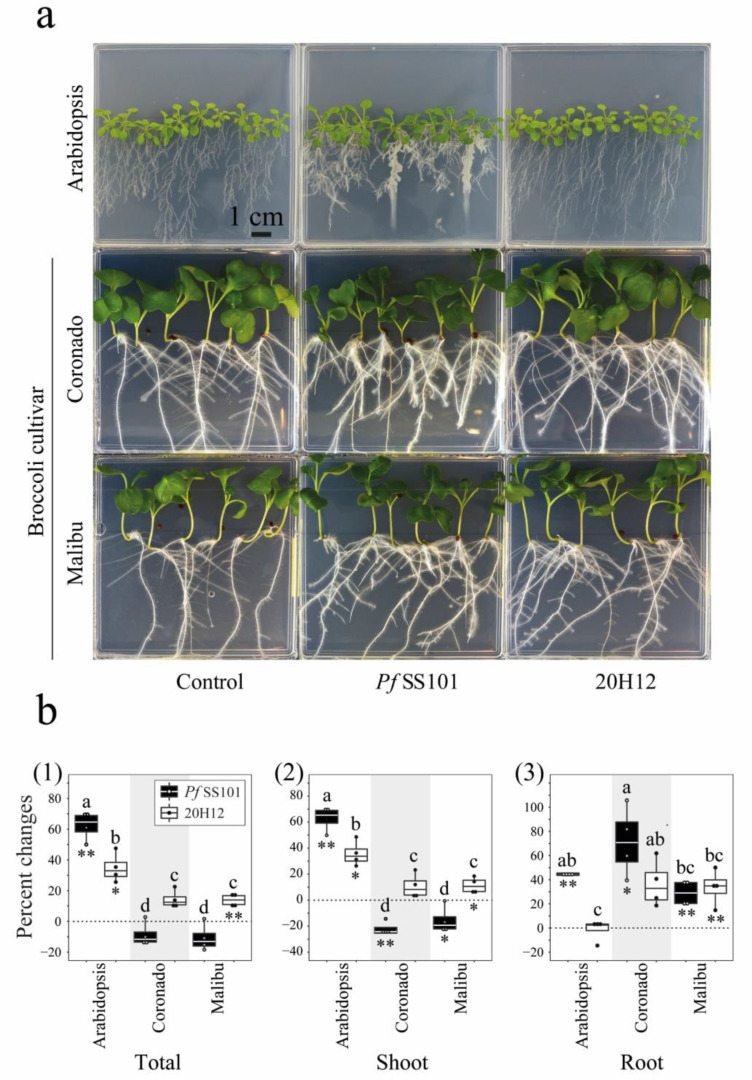
Phenotypic changes in *Arabidopsis* and Broccoli seedlings upon root treatment by *P. fluorescens* SS101 or its *cysH* mutant 20H12. Photographs of MS agar plates with *Arabidopsis*, and two Broccoli cultivars (Coronado and Malibu) treated on the root tip with cell suspensions of *Pf*SS101 (wild type) or its *cysH* mutant (20H12) (**a**). Percentage change in total fresh biomass (**1**), shoot (**2**), and root (**3**) of plants treated with rhizobacteria when compared to untreated plants (11dpi) (**b**). Means of percent changes in biomass with a different letter are significantly different among treatments according to two-way ANOVA (Tukey, *p* < 0.05). Asterisks denote statistically significant differences (two-tailed Student’s *t* test): * *p* < 0.05; ** *p* < 0.01 of rhizobacteria treated plants when compared to the controls. For each plant species, four independent biological replicates were used with 10 seedlings of *Arabidopsis* and five of Broccoli per biological replicate. *Pf*SS101: *Pseudomonas fluorescens* SS101, 20H12: *cysH* mutant of *Pf*SS101.

Treatment of *Arabidopsis* roots with *Pf*SS101 and the *cysH*-mutant 20H12 led to a significant increase in total plant biomass by 62.8% ± 4.6 and 35.1% ± 4.8, respectively (Figure 1(a,b1)). The magnitude of the growth promotion by mutant 20H12 was significantly less than that induced by the wild type. *Pf*SS101 also significantly increased root biomass (44.5% ± 0.6) and changed root architecture by reducing the length of the primary root and enhancing lateral root formation. In contrast, the *cysH*-mutant 20H12 neither affected neither root biomass nor exerted major effects on root architecture (Figure 1(a,b3)). 

For both Broccoli cultivars, the impact of *Pf*SS101 and mutant 20H12 on plant growth was different from that observed for *Arabidopsis*. There was a significant interaction effect between the Broccoli cultivars and the rhizobacteria (wild type/mutant) for the shoot, the root, and total biomass (Supplementary Table S1). Overall, upon root treatment with *Pf*SS101, the total biomass of both Broccoli cultivars showed no significant changes relative to their untreated controls. However, *Pf*SS101 significantly affected biomass allocation between shoot and roots, with significant reductions of the shoot biomass in both cultivars (−22.1% ± 2.6 and −15.6% ± 5.1 in Coronado and Malibu, respectively), and significant increases in root biomass (71.7% ± 14.2 in Coronado and 29.0% ± 5.0 in Malibu) (Figure 1(b2,b3)). Mutant 20H12 significantly enhanced total biomass of Broccoli cultivar Malibu but not of cultivar Coronado. The change in biomass ratio between shoot and roots was not as apparent for mutant 20H12 as it was for wild-type *Pf*SS101. Compared to wild-type *Pf*SS101, however, the overall biomass of both Broccoli cultivars was significantly higher in the 20H12 mutant treatment. Collectively these results suggest that sulfur assimilation in *Pf*SS101 contributes positively to growth promotion in *Arabidopsis*, whereas in Broccoli it has a neutral to negative effect on growth in a cultivar-dependent manner.

To determine if these differential effects on plant biomass were associated with differences in root colonization, the rhizosphere population densities of *Pf*SS101 and *cysH*-mutant 20H12 were assessed at 11 dpi. The results showed that for both *Arabidopsis* and the two Broccoli cultivars, mutant 20H12 generally established up to 10-fold higher rhizosphere population densities than *Pf*SS101 did. Mutant 20H12 established population densities ranging from 2.0 ± 0.1 × 10^6^ to 5.3 ± 0.3 × 10^6^ CFU mg^−1^ whereas densities of *Pf*SS101 ranged from 2.2 ± 0.3 × 10^5^ to 9.9 ± 0.5 × 10^5^ CFU mg^−1^ of root (Supplementary Table S2). These results indicate that the *cysH* gene adversely affected root colonization of *Arabidopsis* and Broccoli by *Pf*SS101. When the different rhizobacterial densities were plotted against the plant biomass changes relative to their non-treated controls (Supplementary Figure S3), no overall consistent correlation was found—higher rhizobacterial population densities were associated with positive, negative, and no changes in shoot and root biomass of the treated plants.

### 3.2. Role of the cysH Gene of P. fluorescens SS101 in Induced Resistance

**Figure 2:**
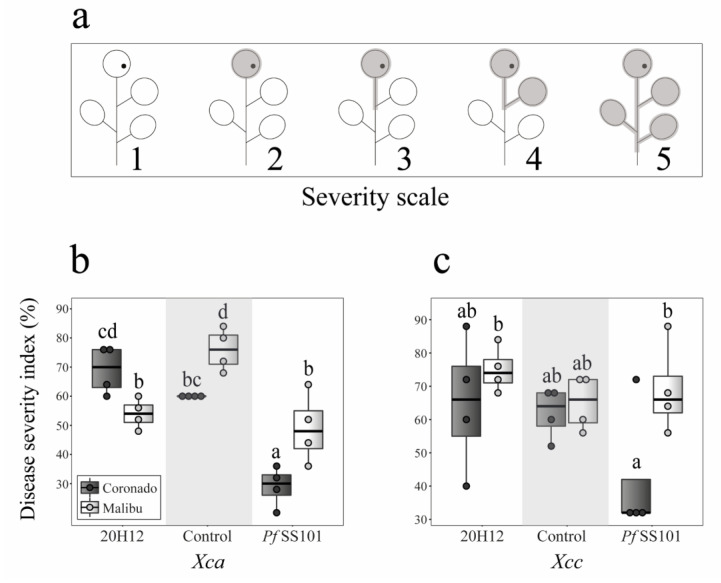
Rhizobacteria-mediated systemic resistance in two Broccoli cultivars, Coronado and Malibu, against the bacterial leaf pathogens. Disease severity was scored on an ordinal scale from 0 to 5, where **1** = no necrosis or migration, **2** = full infection of the treated leaf, **3** = migration of the infection to the leafstalk of the treated leaf, **4** = infection of the neighboring leaf, and **5** = infection of the entire seedling (**a**) (see Supplementary Materials Figure S1 for further details). Details on the conversion of the ordinal scales to disease severity index is provided in the Material and Methods section. Impact of priming roots of Broccoli cultivars with rhizobacteria on severity of leaf disease caused by *Xanthomonas campestris* pv. armoraciae (*Xca*) (**b**) and *Xanthomonas campestris* pv. campestris (*Xcc*) (**c**). Prior to pathogen inoculation on the leaves, roots of each Broccoli cultivar were treated with *P. fluorescens* SS101 or its *cysH*-mutant 20H12 and incubated for 11 days. For the disease severity caused by *Xca* or *Xcc*, Broccoli seedlings from four biological replicates were individually scored (*n* = 20). Different letters above bars indicate statistically significant differences based on beta regression analysis followed by Tukey test (*p* < 0.05).

For the two Broccoli cultivars, we also studied the effect of root inoculation with *Pf*SS101 or its *cysH*-mutant 20H12 on infection of the leaves by two pathovars of the bacterial leaf pathogen *Xanthomonas campestris*, which causes “black rot” in the crucifer family [17]. Broccoli cultivar Coronado pretreated on the roots with *Pf*SS101 and challenged on the leaves with *Xanthomonas campestris* pv. armoraciae (*Xca*) showed significant reduction in disease severity, whereas treatment with mutant 20H12 was ineffective (Figure 2a). By contrast, both *Pf*SS101 and mutant 20H12 induced resistance in cultivar Malibu against *Xca* (Figure 2). However, *Pf*SS101 and mutant 20H12 had no significant impact on *Xcc* disease severity for both Broccoli cultivars (Figure 2b). The results further showed that a higher (qualitative) disease severity index corresponded to a higher cell density of *Xca* in the leaves (Supplementary Figure S4). In contrast, no clear correlation between cell density and disease severity index was observed for *Xcc*.

### 3.3. Effect of P. fluorescens SS101 and the cysH Mutant on the Plant Metabolome 

Non-targeted LC–MS-based metabolite profiling was applied to investigate the impact of the *cysH* mutation in *Pf*SS101 on changes in the shoot secondary metabolome and to identify potential associations with the phenotypic changes induced by these rhizobacteria as described above. A fold-change (FC) cut-off of >2 and significance *p*-values of <0.05 in ANOVA were used to identify metabolites that were altered significantly by the treatment of the plants root with *Pf*SS101 or its *cysH* mutant and used to compute principal component analysis (PCA) and hierarchical cluster analysis (HCA).

#### 3.3.1. *Arabidopsis*

**Figure 3:**
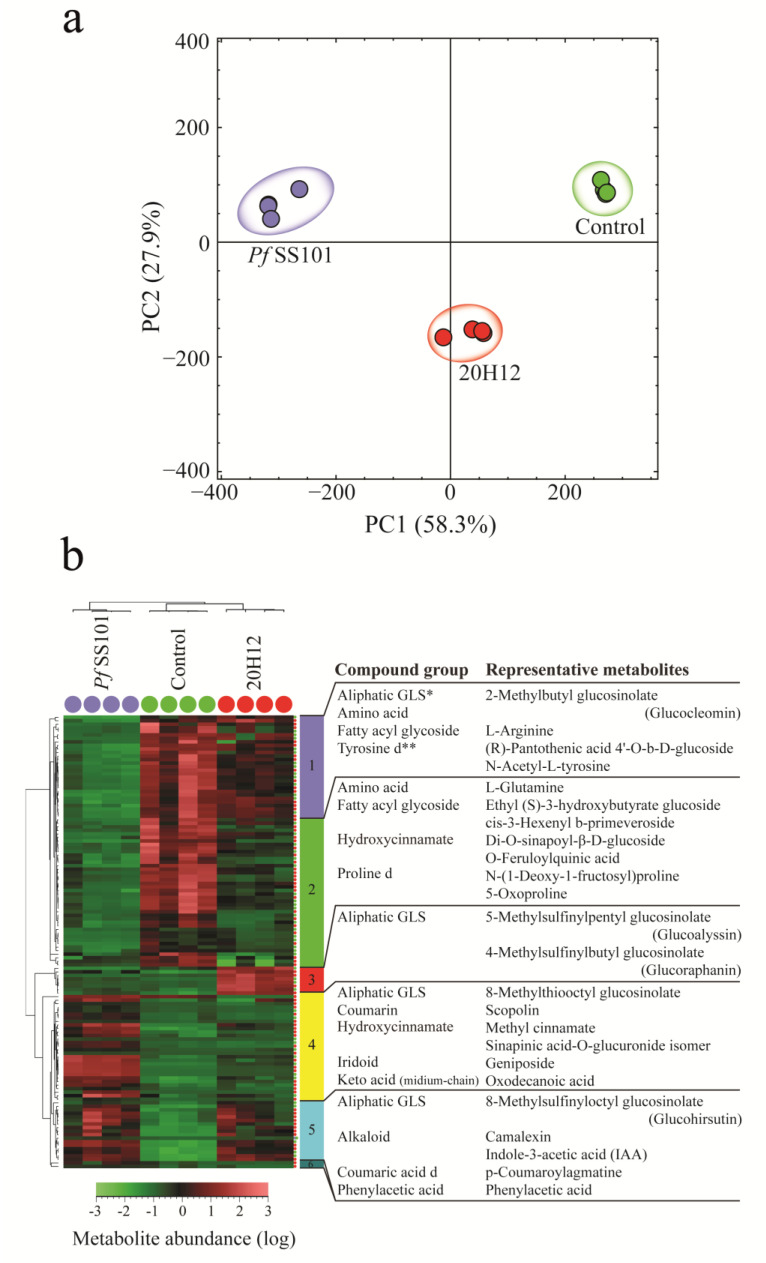
Metabolome changes in *Arabidopsis* shoots upon root treatment by *P. fluorescens* SS101 or its *cysH* mutant 20H12. Shown are the results of the principal component analysis (PCA) (**a**) and hierarchical cluster analysis (HCA) (**b**) based on the differentially regulated semi-polar metabolites. In the HCA, various metabolite clusters are indicated by different colors and when none of the metabolites in a given cluster was annotated, the cluster number was omitted (clusters 3, 5, and 10 in panel b). * GLS, glucosinolate; ** d, derivative.

From a total of 725 metabolites detected in *Arabidopsis* shoots in either positive or negative ionization mode, 128 (18%) metabolites were significantly different between the *Pf*SS101 and its *cysH*-mutant treatments. Abundance and fold changes of each detected plant metabolite are shown in Supplementary Materials, Table S6. PCA of the samples based on the relative abundance of the 725 metabolites revealed a clear discrimination between both treatments with the first two principal components explaining 86% of the total variance (Figure 3a). The first principal component (PC1) accounted for 58.3% of the total variance and was associated with metabolites showing a contrasting accumulation pattern between *Pseudomonas* (*Pf*SS101 or 20H12)-treated and untreated plants. The HCA plot (Figure 3b) showed metabolites that were either depleted or increased in plants treated with *Pf*SS101 or 20H12 as compared to the controls (Figure 3b, clusters 1–2 (depleted) and clusters 4–5 (accumulated)). Metabolites in cluster 1 showed significant depletion in plants treated with *Pf*SS101 and include the short-chain (C-4) isoleucine-derived aliphatic 2-methylbutyl GLS (glucocleomin), amino acids, and derivatives such as arginine and N-acetyl-L-tyrosine, and fatty acyl glycosides such as pantothenic acid-glucoside. Cluster 2 comprises metabolites that were depleted in plants treated with both *Pf*SS101 and the mutant 20H12, and the depletion was more pronounced on plants treated with *Pf*SS101. Some of the identified metabolites in this cluster include an amino acid and derivatives thereof such as glutamine and N-(1-deoxy-1-fructosyl) proline, 5-oxoproline, fatty acyl glycosides, and O-feruloylquinic acid. Metabolites that were increased by the *Pf*SS101 treatment are shown in Cluster 4 and include a long-chain (C-8) aliphatic-GLS, i.e., 8-methylthiooctyl glucosinolate, a hydroxycinnamic acid glucuronide, i.e., sinapic acid-O-glucuronide, the medium-chained keto acid oxodecanoic acid, and the coumarin scopolin (see cluster 4). Cluster 5 represents metabolites that were enhanced in both *Pf*SS101 and 20H12 treatments and include the long-chain (C-8) aliphatic-GLS 8-methylsulfinyloctyl glucosinolate (glucohirsutin), the hydroxycinnamic acid amide coumaroylagmatine and stress-associated alkaloids such as camalexin (20.5-fold up in *Pf*SS101 and 17.3-fold up in 20H12), and indole-3-acetic acid (IAA) (8.4-fold in *Pf*SS101 and 6.9-fold in 20H12). The second principal component (PC2) explained 27.9% of the total variance and includes metabolites that were mainly accumulated in plants treated with the 20H12 (Cluster 3) relative to the other treatments. Here, 4-methylsulfinylbutyl GLS (glucoraphanin) and 5-methylsulfinylpentyl GLS (glucoalyssin), middle-chained (C-4 and C-5, respectively) aliphatic-GLSs, displayed 20H12-specific accumulation.

In conclusion, the most prominent similarities in shoot metabolome changes induced in *Arabidopsis* shoots by *Pf*SS101 and mutant 20H12 are enhanced levels of the plant growth hormone IAA and the stress-associated alkaloid camalexin, while the most prominent differences in shoot metabolome involve the medium-chain keto acid oxodecanoic acid, and the sulfur-containing compounds 8-methylthiooctyl, 4-methylsulfinylbutyl, and 5-methylsulfinylpentyl GLS; these were all less enhanced in plants colonized by the *cysH* mutant compared to plants colonized by wild-type *Pf*SS101.

#### 3.3.2. Broccoli

For Broccoli, two cultivars were selected based on their contrasting levels of GLSs (glucoiberin, glucoraphanin, and glucobrassicin) and thereby possible differences in sulfur needs. Our metabolome analysis confirmed that cultivar Coronado indeed has higher levels of these three GLSs (glucoiberin, glucoraphanin, and glucobrassicin) than cultivar Malibu (Table S5). Treatment of the roots of Coronado and Malibu with *Pf*SS101 further enhanced the levels of the indole GLS glucobrassicin by 5- and 4-fold, respectively, whereas the levels of the other two aliphatic GLSs were not significantly changed upon *Pf*SS101 treatment (Table S5). Treatment of the roots of both cultivars with the *cysH* mutant did not enhance the levels of these three GLSs or at least not to the same levels as observed for wild-type *Pf*SS101 (Table S5).

**Figure 4:**
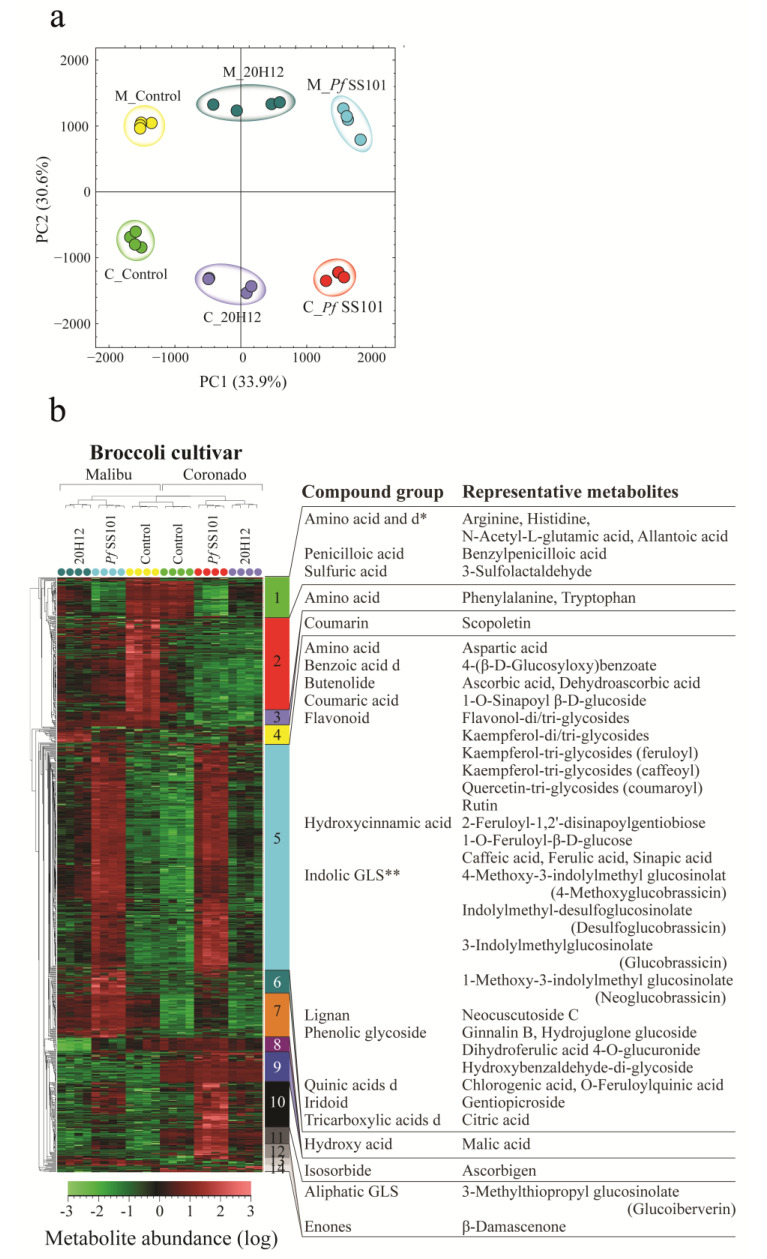
Metabolome changes in the shoots of two Broccoli cultivars, Coronado and Malibu upon root treatment by *P. fluorescens* SS101 or its *cysH* mutant 20H12. Shown are the results of the PCA (**a**) and HCA (**b**) based on differentially regulated semi-polar metabolites. In the HCA, various metabolite clusters are indicated by different colors; when none of the metabolites in a given cluster was annotated, the cluster number was omitted (clusters 3, 6, 8, 9, 12, 13, and 14 in panel b). * GLS, glucosinolate; ** d, derivative.

The *cysH* mutation in *Pf*SS101 did have substantial impact on the overall Broccoli shoot metabolism (Figure 4). Detailed information about abundance and fold change of each metabolite is provided in Supplementary Materials, Table S7. From a total of 1908 metabolites detected in Broccoli shoot samples, 830 (44%) metabolites were significantly different between the *Pf*SS101 and 20H12 treatments. In the PCA, the first two PCs explained 64% of the total variation. The first PC that explained 33.9% of the total variation corresponds to the metabolites that showed a contrasting accumulation pattern between inoculated and un-inoculated Broccoli plants with *Pseudomonas* (*Pf*SS101 or mutant 20H12). The HCA plot (Figure 4b) showed metabolites that were depleted or increased in plants treated with *Pf*SS101 or 20H12 as compared to the controls. The magnitude of the alteration of these metabolites was greater in *Pf*SS101 treatment (i.e., cluster 5: 307 metabolites; cluster 1: 55 metabolites). Cluster 5, the largest cluster of *Pf*SS101-enhanced metabolites, comprised metabolites associated with plant-defense-related biosynthesis of phenolic compounds such as the flavonoids kaempferol-di/tri-(feruloyl/ caffeoyl/ coumaroyl) glycosides, quercetin-tri-coumaroyl glycoside, rutin, hydroxycinnamates caffeic acid, ferulic acid, feruloylquinic acid, sinapic acid, chlorogenic acid, neocuscutoside C, and their derivatives, and resveratrol sulfoglucoside. In addition, some phenolic glucosides including ginnalin B, hydroxybenzaldehyde diglycoside, and hydrojuglone glucoside, as well as antioxidants such as ascorbic acid (vitamin C) and dehydroascorbic acid also belonged to this metabolite cluster that was enhanced in the *Pf*SS101 treatment. Among the identified GLSs in cluster 5, the indole-GLS glucobrassicin and its derivatives showed significant increases in the *Pf*SS101 treatment.

Cluster 1 encompasses metabolites that were reduced in plants inoculated with *Pf*SS101 and includes some amino acids and derivatives, allantoic acid, and benzylpenicilloic acid, as well as 3-sulfolactaldehyde. PC2 explained 30.6% of the total variation and was associated with metabolites that were intrinsically different in abundance between the two Broccoli cultivars (Clusters 2 and 7 (high in Malibu) and clusters 9 and 11 (high in Coronado)). Furthermore, metabolite clusters 7, 9, and 11 showed higher accumulation in plants treated with *Pf*SS101 when compared to mutant 20H12. Metabolites in both Broccoli cultivars that were similarly decreased by *Pf*SS101 and *cysH* mutant, as compared to the control, grouped together in cluster 2 and included phenylalanine and tryptophan, which are the building blocks for phenylpropanoid and indole-GLS biosynthesis, respectively. In addition, malic acid was most abundant (1.8-fold) in Malibu (cluster 7) while the level of the aliphatic-GLS glucoiberverin was much higher (92-fold) in Coronado (cluster 11). The third PC explained 9.2% of the total variation (not shown) and was associated with metabolites that accumulated (cluster 4) or decreased in Malibu (cluster 8) treated with 20H12. In summary, the *cysH* mutation in *Pf*SS101 specifically affected the abundance of metabolites in Broccoli shoots associated with flavonoid, hydroxycinnamate, and indole-GLS biosynthesis.

## 4. Discussion and Conclusions

Sulfur is essential for plant growth and plays an important role in plant defense against fungal pathogens and insect pests. We previously showed that the rhizobacterium *Pf*SS101 promoted growth and altered root architecture of *Arabidopsis*, induced systemic resistance (ISR) and, based on targeted metabolomics, enhanced GLS levels in both roots and shoots [8]. By screening a genome-wide mutant library of *Pf*SS101, we then identified the adenylsulfate reductase gene *cysH* as one of the key genes associated with growth promotion and ISR in *Arabidopsis* [4]. *cysH* is involved in sulfur assimilation and the biosynthesis of cysteine and methionine. Results from Cheng et al. [4] further showed that addition of cysteine to the growth medium induced lateral root formation in *Arabidopsis* in a concentration-dependent manner and triggered ISR against the bacterial leaf pathogen *Pseudomonas syringae*. Cysteine can enter the GLS biosynthesis pathway of *Arabidopsis* by different routes including (i) direct donation of reduced sulfur needed for GLS biosynthesis, (ii) incorporation of cysteine into methionine that, through a series of side-chain elongations, S-glycosylation, and other secondary modifications, ends up in the GLS pool, and (iii) conjugation of cysteine, glutamate, and glycine to form glutathione (GSH) [19] which in turn can act as a sulfur donor in GLS biosynthesis [20]. The results of our study confirmed the earlier observation that cysteine-derived GLS levels were increased in shoots of *Arabidopsis* upon root treatment with *Pf*SS101 and provided several new findings. These include higher levels of two octyl GLS forms, i.e., 8-methylthio and 8-methylsulfinyl, in plants colonized by *Pf*SS101. Furthermore, the isoleucine-derived short-chain (C-4) aliphatic 2-methylbutyl GLS showed significant reduction in plants treated with *Pf*SS101. In *Arabidopsis*, side-chain elongation of aliphatic-GLS is catalyzed by the MAM1/MAM3 proteins, which condense 2-oxo acids and acetyl-CoA to extend the alkane C chain [21,22]. Oxodecanoic acid, which is a potential substrate for chain elongation, also showed greater accumulation in plants treated with *Pf*SS101. Interestingly, transcriptome analysis from our previous study revealed that plants treated with *Pf*SS101 showed significantly higher expression of both MAM1 and MAM3 genes when compared with plants treated with the *cysH* mutant or compared with untreated plants [4]. Relative to *Pf*SS101-treated plants, *cysH*-mutant-treated *Arabidopsis* seedlings contained lower levels of oxodecanoic acid and the long-chain octyl GLSs, and higher levels of the short-chain 4-methylsulfinylbutyl glucosinolate (C4) and 5-methylsulfinylpentyl glucosinolate (C5). Collectively these results indicate that the *cysH* gene of *Pf*SS101 affects chain elongation of aliphatic-GLSs in leaves of *Pf*SS101-treated *Arabidopsis*. 

Sulfur is essential for plant growth and plays an important role in plant defense against fungal pathogens and insect pests. We previously showed that the rhizobacterium *Pf*SS101 promoted growth and altered root architecture of *Arabidopsis*, induced systemic resistance (ISR) and, based on targeted metabolomics, enhanced GLS levels in both roots and shoots [8]. By screening a genome-wide mutant library of *Pf*SS101, we then identified the adenylsulfate reductase gene *cysH* as one of the key genes associated with growth promotion and ISR in *Arabidopsis* [4]. *cysH* is involved in sulfur assimilation and the biosynthesis of cysteine and methionine. Results from Cheng et al. [4] further showed that addition of cysteine to the growth medium induced lateral root formation in *Arabidopsis* in a concentration-dependent manner and triggered ISR against the bacterial leaf pathogen *Pseudomonas syringae*. Cysteine can enter the GLS biosynthesis pathway of *Arabidopsis* by different routes including (i) direct donation of reduced sulfur needed for GLS biosynthesis, (ii) incorporation of cysteine into methionine that, through a series of side-chain elongations, S-glycosylation, and other secondary modifications, ends up in the GLS pool, and (iii) conjugation of cysteine, glutamate, and glycine to form glutathione (GSH) [19] which in turn can act as a sulfur donor in GLS biosynthesis [20]. The results of our study confirmed the earlier observation that cysteine-derived GLS levels were increased in shoots of *Arabidopsis* upon root treatment with *Pf*SS101 and provided several new findings. These include higher levels of two octyl GLS forms, i.e., 8-methylthio and 8-methylsulfinyl, in plants colonized by *Pf*SS101. Furthermore, the isoleucine-derived short-chain (C-4) aliphatic 2-methylbutyl GLS showed significant reduction in plants treated with *Pf*SS101. In *Arabidopsis*, side-chain elongation of aliphatic-GLS is catalyzed by the MAM1/MAM3 proteins, which condense 2-oxo acids and acetyl-CoA to extend the alkane C chain [21,22]. Oxodecanoic acid, which is a potential substrate for chain elongation, also showed greater accumulation in plants treated with *Pf*SS101. Interestingly, transcriptome analysis from our previous study revealed that plants treated with *Pf*SS101 showed significantly higher expression of both MAM1 and MAM3 genes when compared with plants treated with the *cysH* mutant or compared with untreated plants [4]. Relative to *Pf*SS101-treated plants, *cysH*-mutant-treated *Arabidopsis* seedlings contained lower levels of oxodecanoic acid and the long-chain octyl GLSs, and higher levels of the short-chain 4-methylsulfinylbutyl glucosinolate (C4) and 5-methylsulfinylpentyl glucosinolate (C5). Collectively these results indicate that the *cysH* gene of *Pf*SS101 affects chain elongation of aliphatic-GLSs in leaves of *Pf*SS101-treated *Arabidopsis*. 

For Broccoli, *cysH*-mediated sulfur metabolism in *Pf*SS101 appeared to adversely affect shoot biomass, as in both cultivars root treatment with the *cysH* mutant resulted in higher overall biomass compared to wild-type *Pf*SS101. The total biomass of both *Pf*SS101-treated cultivars showed no significant changes relative to their non-treated controls, but *Pf*SS101 significantly affected biomass allocation between shoot and roots with significant reductions of the shoot biomass and significant increases in the root biomass. In contrast to wild-type *Pf*SS101, the *cysH* mutant significantly enhanced total biomass of cultivar Malibu. Also, the change in biomass allocation by the *cysH* mutant was not as apparent as it was for wild-type *Pf*SS101. Collectively these results suggest that *cysH*-mediated sulfur assimilation in *Pf*SS101 has a neutral to negative effect on Broccoli plant growth in a cultivar-dependent manner. In the Broccoli shoot metabolome, *Pf*SS101 enhanced the accumulation of defense-associated metabolites, specifically those related to the phenolic pathway of flavonols, including kaempferol and quercetin glycosides and various hydroxycinnamates, such as caffeic acid and ferulic acid conjugates, as well as the antioxidant ascorbic acid and the indole-GLSs glucobrassicin, desulfoglucobrassicin (indolylmethyl-desulfoglucosinolate), and 4-methoxyglucobrassicin (4-methoxy-3-indolylmethyl glucosinolate). By contrast, the levels of these flavonoids, hydroxycinnamates, and indole-GLSs only showed slight to moderate increases in Broccoli seedlings treated with the *cysH* mutant. The enhanced level of glucobrassicin and its derivatives, and the concomitant lower shoot biomass in *Pf*SS101-treated seedlings suggest that *cysH*-mediated sulfur assimilation in *Pf*SS101 can modulate tryptophan metabolism in Broccoli towards the biosynthesis of indole-GLSs instead of the growth-promoting phytohormone IAA. Furthermore, the relatively high abundance of flavonoids in Broccoli plants treated with *Pf*SS101 could impact auxin biosynthesis, transport [23], and distribution and its conjugation/degradation [24], thereby affecting plant growth. Additionally, the higher accumulation of phenylpropanoids and other secondary metabolites in Broccoli seedlings treated with *Pf*SS101 could pose a limitation of carbon and energy resources, leading to an adverse effect on plant growth. 

Although sulfur assimilation by *Pf*SS101 adversely affected overall growth in Broccoli, it led to enhanced defense against *Xca* in a cultivar-specific manner. When roots of the cultivar Coronado were treated with *Pf*SS101 and the leaves challenged with *Xca*, a significantly reduced disease severity was observed, whereas root treatment with *cysH*-mutant 20H12 was not effective. By contrast, in cultivar Malibu both *Pf*SS101 and mutant 20H12 reduced disease severity caused by *Xca*. The extent of *Pf*SS101-mediated changes in the Broccoli shoot metabolome, particularly towards defensive secondary metabolites such as flavonoids, hydroxycinnamates, lignin, iridoid glycosides, and indole-GLSs was substantial. Phenolic compounds can have direct or indirect inhibitory effect on bacterial pathogens, involving disruption of growth- and reproduction-related processes in the pathogens [25,26], while the indirect effect involves limiting the pathogen ingress by fortifying the plant cell wall [27,28]. Similarly, the indole-GLS glucobrassicin was reported to have inhibitory activity against *Xcc* in *Brassica oleracea* [29] while 4-methoxyglucobrassicin was implicated as a signal molecule in plant defense against both bacteria and fungi [30]. Hence, *Pf*SS101-mediated systemic resistance in Broccoli against *Xca* and to some extent against *Xcc* is likely related, at least partly, to the observed changes in these shoot secondary metabolites. These changes in the shoot metabolome are likely associated with pathogen defense and their impact is dependent on the pathovar (*Xca*/*Xcc*) of the pathogen.

Interestingly, there were other metabolite changes observed in Broccoli shoots that appear to be disconnected from *cysH*-mediated sulfur assimilation in *Pf*SS101. For example, the level of ascorbic acid (vitamin C), an antioxidant element [31,32], was enhanced in *Pf*SS101-treated seedlings as well, possibly by an upregulation of the L-galactose pathway of ascorbate biosynthesis or due to less oxidative stress. In addition, a significant increase of scopolin, a coumarin glycoside, was detected in our study. Recent work on *Arabidopsis* treated with growth-promoting *Pseudomonas simiae* strain WCS417 revealed enhanced levels of scopoletin in root exudates and showed that scopoletin is an iron-mobilizing phenolic compound with selective antimicrobial activity that shapes the root-associated microbial community [33]. Our results show that scopolin also accumulated in the leaves upon root treatment with the plant-growth-promoting *Pseudomonas* strain *Pf*SS101. Whether these coumarins also have antibacterial activity against the two *Xanthomonas campestris* pathovars of Broccoli tested in this study, will be the subject of future investigations to unravel the importance of coumarins in the observed ISR response.

In conclusion, our results showed, for the first time, that disruption of sulfur metabolism in root-colonizing *Pseudomonas* exerts significant changes in host plant secondary metabolism, in particular chain elongation of aliphatic glucosinolates (GLSs) and indole metabolites, including camalexin and the growth hormone indole-3-acetic acid. In Broccoli, *Pf*SS101 sulfur assimilation significantly upregulated the relative abundance of several shoot metabolites, in particular indolic GLSs and phenylpropanoids, metabolome changes that coincided with *Pf*SS101-mediated suppression of leaf infections by *Xanthomonas campestris*. Our study provides evidence of the interconnectedness of the metabolism of the host and that of a member of their root-associated microbiota. Integration of metabolomics with transcriptomics data followed by targeted mutagenesis or silencing of specific genes or pathways in the host plant will be needed to validate the functional importance of the observed metabolome changes for plant growth and defense.

## Data Availability

Not applicable.

## Supplementary Materials

The following are available online at https://www.mdpi.com/article/10.3390/biom11111704/s1, Figure S1: Disease severity scale used to assess the level of induced resistance in Broccoli at 11 dpi., Figure S2: Biomass changes (absolute) of the whole plant, shoot, and root after rhizobacteria treatment (11 dpi)., Figure S3: Correlation between host phenotypic changes and root colonization of *Pseudomonas fluorescens* SS101 and *cysH* mutant 20H12, Figure S4: Correlation between disease severity of Broccoli and cell density of the bacterial pathogen *Xca* (A): *Xanthomonas campestris* pv. armoraciae, and *Xcc* (B): *Xanthomonas campestris* pv. campestris., Table S1: Results of the analysis of variance ANOVA (type II) of phenotypic changes induced in two Broccoli cultivars by root inoculation by *Pseudomonas fluorescens* strain SS101 or its *cysH* mutant 20H12., Table S2: Population density of *Pseudomonas fluorescens* SS101 and *cysH*-mutant 20H12 on roots of *Arabidopsis* and two Broccoli cultivars, Coronado and Malibu, at 11 dpi., Table S3: Effects of root treatment of two Broccoli cultivars with the beneficial rhizobacterium *Pseudomonas fluorescens* SS101, or with its *cysH* mutant on the phylosphere population density of two pathovars of the bacterial leaf pathogen Xanthomonas campestris at 11 dpi., Table S4: Results of the beta-regression analysis of disease severity caused by *Xanthomonas* on leaves two Broccoli cultivars (Coronado and Malibu) that were pretreated on the roots with *Pseudomonas fluorescens* SS101 or its *cysH* mutant., Table S5: Level of three glucosinolates (GLSs: glucoiberin, glucoraphanin and glucobrassicin) in the shoot of the two Broccoli cultivars, Coronado and Malibu, Table S6: Annotation of *Arabidopsis* shoot metabolites that showed significant change in their abundance after root inoculation with rhizobacterium *Pf*SS101: *Pseudomonas fluorescens* SS101, or its mutant 20H12: *cysH* mutant of *Pf*SS101., Table S7: Annotation of Broccoli shoot metabolites that showed significant change in their abundance after root inoculation with rhizobacterium *Pf*SS101: *Pseudomonas fluorescens* SS101, or its mutant 20H12: *cysH* mutant of *Pf*SS101.
