# Supplementary material for: Effects of Sulfur Assimilation in Pseudomonas fluorescens SS101 on Growth, Defense, and Metabolome of Different Brassicaceae
Source: Biomolecules. 2021 Nov 16;11(11):1704. doi: 10.3390/biom11111704 (PMC8615669; doi:10.3390/biom11111704)
Supplement: Supplementary file 1 [file biomolecules-11-01704-s001.zip › biomolecules-1410424-rev done-supplementary-figures.pdf]

Supplementary figures

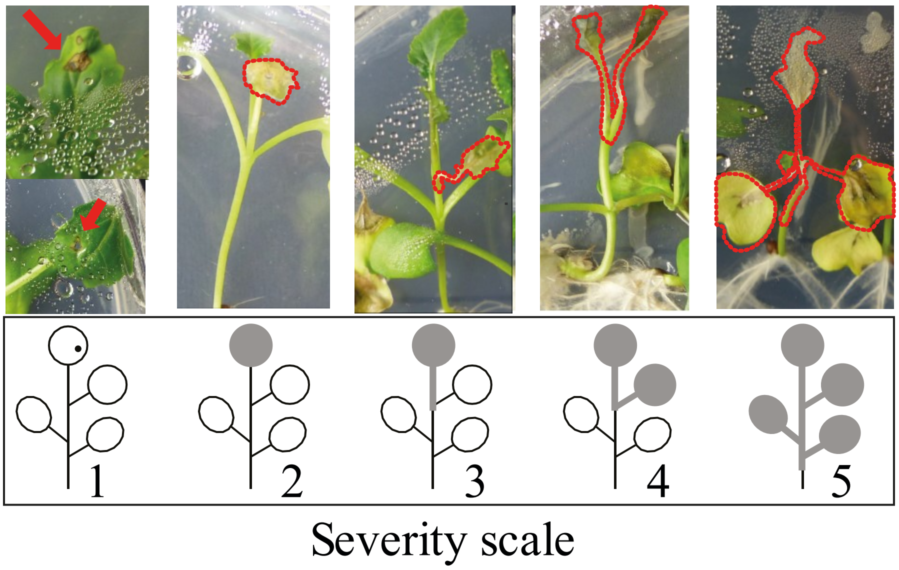

Figure S1. Disease severity scale used to assess the level of induced resistance in Broccoli at 11 dpi. 1 = no necrosis, no migration of the infection symptom of the bacterial leaf pathogen *Xanthomonas campestris*, 2 = dispersal of the infection symptom over the treated leaf, 3 = migration of the infection symptom to the leafstalk of the treated leaf, 4 = migration to the neighboring leaf, and 5 = migration to the entire seedlings after inoculation.

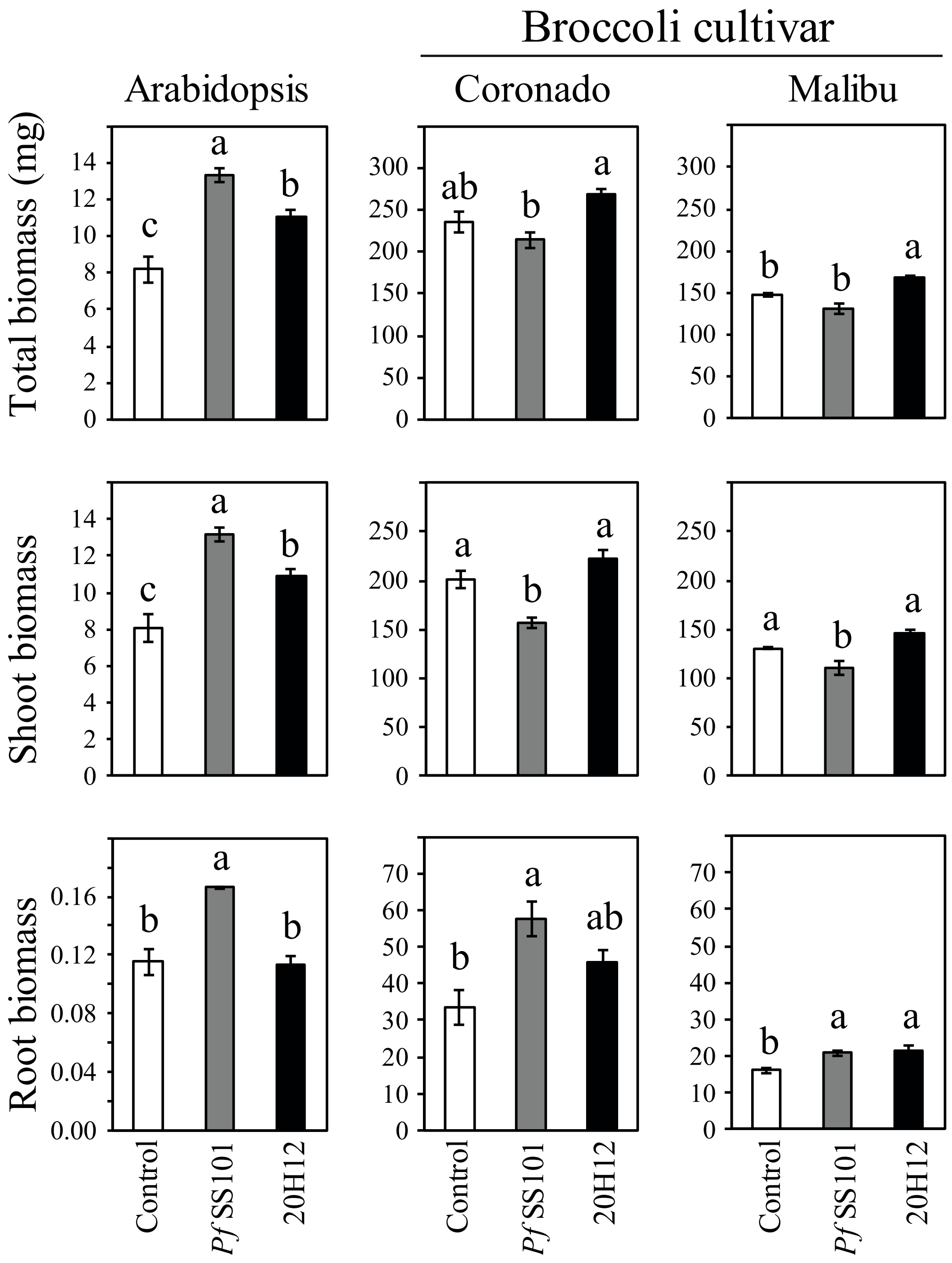

Figure S2. Rhizobacteria-mediated changes in biomass in different Brassicaceae. The recorded total, shoot and root biomass (mg) for the different plants after rhizobacteria treatment (11 dpi) are shown in the y axis. Bars with different letters are significantly different between the treatments according to one-way ANOVA (Tukey, P<0.05). For each plant species, four independent biological replicates were used with 10 seedlings of Arabidopsis and 5 of Broccoli per replicate. *Pf*SS101: *Pseudomonas fluorescens* SS101, 20H12: *cysH* gene mutant of *Pf*SS101.

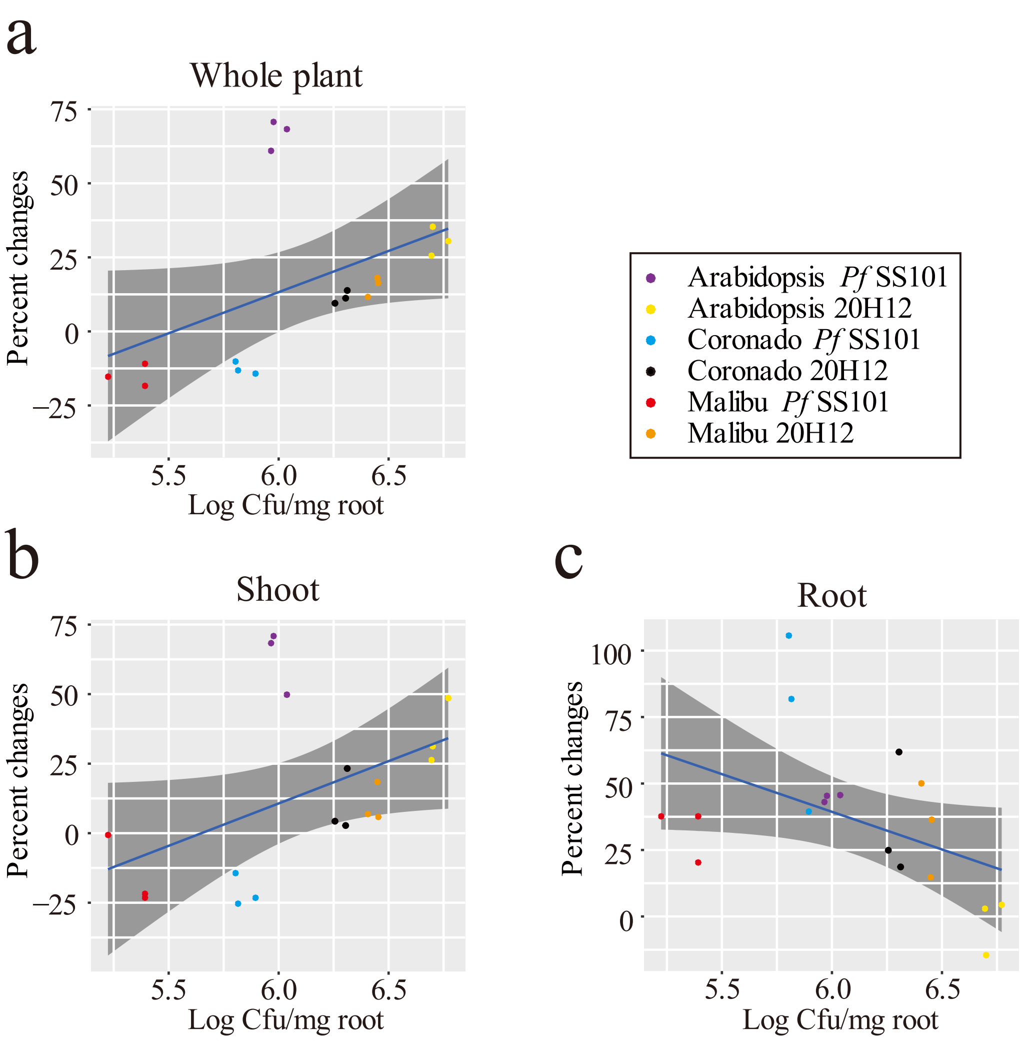

Figure S3. Correlation between change in host biomass and root colonization of *Pseudomonas fluorescens* SS101 and *cysH* mutant 20H12. Correlation between the rhizosphere population density of rhizobacteria (Log Cfu/mg root) and percent changes of biomass of whole plant **(a)**, shoot **(b)** and root **(c)** of Arabidopsis and two Broccoli cultivars: Coronado and Malibu at 11dpi. *Pf*SS101: *Pseudomonas fluorescens* SS101, 20H12: *cysH* mutant of *Pf*SS101. In the graphs, the grey zone surrounding the regression line represents the 95% confidence interval.

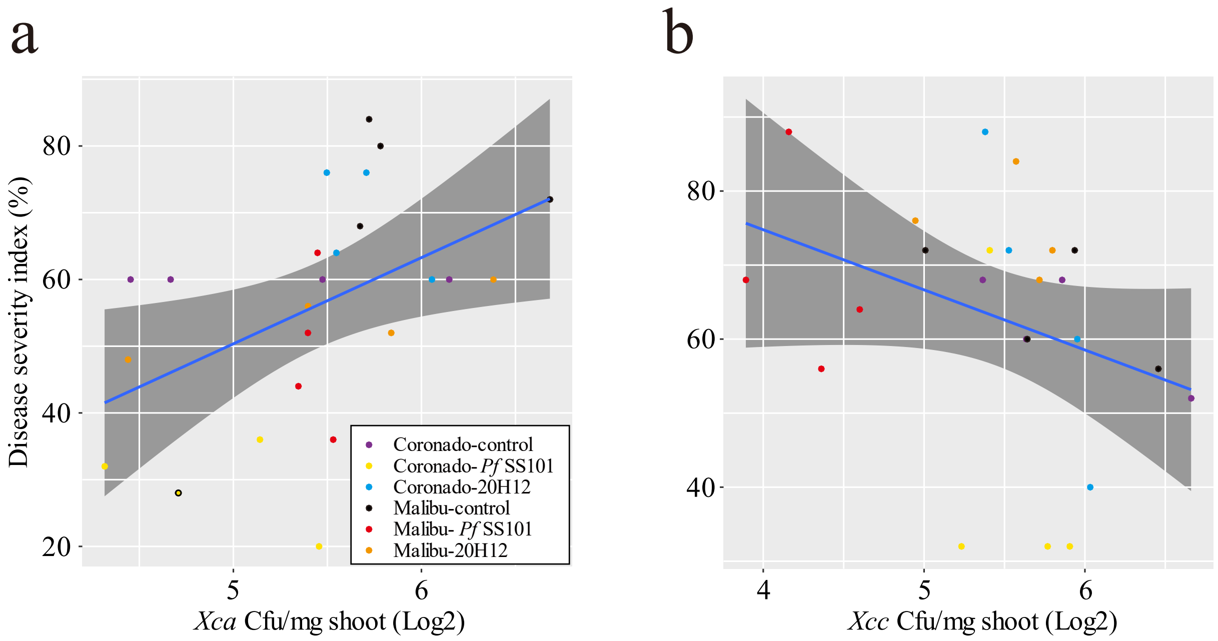

Figure S4. Correlation between disease severity of Broccoli seedlings and cell density of the bacterial pathogen *Xca* (**a**): *Xanthomonas campestris* pv. armoraciae, and *Xcc* (**b**): *Xanthomonas campestris* pv. campestris. C: Coronado, M: Malibu, Control: Broccoli without bacteria treatment, *Pf*SS101: *Pseudomonas fluorescens* SS101; 20H12: *cysH*-mutant of *Pf*SS101. In the graphs, the grey zone surrounding the regression line represents the 95% confidence interval.
