# Supplementary material for: Effects of Sulfur Assimilation in Pseudomonas fluorescens SS101 on Growth, Defense, and Metabolome of Different Brassicaceae
Source: Biomolecules. 2021 Nov 16;11(11):1704. doi: 10.3390/biom11111704 (PMC8615669; doi:10.3390/biom11111704)
Supplement: Supplementary file 1 [file biomolecules-11-01704-s001.zip › biomolecules-1410424-rev done-supplementary-tableS1-5.pdf]

Supplementary tables

Table S1. Results of the analysis of variance ANOVA (type II) of phenotypic changes induced in tow Broccoli cultivars by root inoculation by *Pseudomonas fluorescens* strain SS101 or its *cysH* mutant 20H12. Shown are whole plant, shoot and root biomass changes in the two broccoli cultivars after root inoculation with *Pf*SS101: *Pseudomonas fluorescens* SS101, 20H12: *cysH*-mutant of *Pf*SS101.

Sample	Factor	Df	Sum Sq	Mean Sq	F value	Pr(>F)	
Whole plant fresh biomass relative change	Broccoli cultivars	2	11513	5757	94.559	2.83E-10	***
	Bacteria (WT vs Mutant)	1	260	260	4.263	0.0537	.
	Broccoli cultivars: Bacteria	2	3536	1768	29.043	2.32E-06	***
	Residuals	18	1096	61			
Shoot fresh biomass relative change	Broccoli cultivars	2	15124	7562	103.416	1.35E-10	***
	Bacteria	1	699	699	9.554	0.0063	**
	Broccoli cultivars: Bacteria	2	4373	2186	29.899	1.90E-06	***
	Residuals	18	1316	73			
Root fresh biomass relative change	Broccoli cultivars	2	4453	2226	8.477	0.00255	**
	Bacteria	1	3883	3883	14.783	0.00119	**
	Broccoli cultivars: Bacteria	2	2829	1415	5.386	0.01468	*
	Residuals	18	4728	263			

Significance codes: *** 0, ** 0.001, * 0.01.

Table S2. Population density of *Pseudomonas fluorescens* SS101 and *cysH*-mutant 20H12 on roots of Arabidopsis and two Broccoli cultivars, Coronado and Malibu, at 11 dpi. *Pf*SS101: *Pseudomonas fluorescens* SS101, 20H12: *cysH*-mutant of *Pf*SS101.

Plant species		Population density (Cfu/mg*)	
		*Pf*SS101	20H12
Arabidopsis thaliana Col-0		$9.9 \pm 0.5 \times 10^5$	$5.3 \pm 0.3 \times 10^6$
Brassica oleracea var. *italica*	Coronado	$6.9 \pm 0.5 \times 10^5$	$2.0 \pm 0.1 \times 10^6$
	Malibu	$2.2 \pm 0.3 \times 10^5$	$2.7 \pm 0.1 \times 10^6$

CFU: Colony forming unit

*Values represent the average of 3 replicates ± SE of three replicates

Table S3. Effects of root treatment of two Broccoli cultivars with the beneficial rhizobacterium *Pseudomonas fluorescens* SS101, or with its *cysH*-mutant on the phylosphere population density of two pathovars of the bacterial leaf pathogen *Xanthomonas campestris* at 11 dpi. The two Broccoli cultivars are Coronado and Malibu; the two pathovars of *X. campestris* are *Xca*: *X. campestris* pv. armoraciae, and *Xcc*: *X. campestris* pv. campestris

Broccoli cultivars	Rhizobacteria pretreatments	Population density (Cfu/mg*)	
		Xca	*Xcc*
Coronado	Control	$4.4 \pm 3.3 \times 10^5$	$1.5 \pm 1.0 \times 10^6$
	*Pf*SS101	$1.2 \pm 0.6 \times 10^5$	$4.5 \pm 1.5 \times 10^5$
	20H12	$5.8 \pm 1.9 \times 10^5$	$6.4 \pm 2.1 \times 10^5$
Malibu	Control	$1.6 \pm 1.1 \times 10^6$	$1.1 \pm 6.2 \times 10^6$
	*Pf*SS101	$2.7 \pm 0.3 \times 10^5$	$2.1 \pm 0.1 \times 10^4$
	20H12	$8.5 \pm 5.4 \times 10^5$	$4.0 \pm 1.2 \times 10^5$

Cfu: Colony forming unit
*Values represent the average of 3 replicates ± SE of three replicates

Table S4. Results of the beta regression analysis of disease severity caused by *Xanthomonas* on leaves two Broccoli cultivars (Coronado and Malibu) that were pretreated on the roots with *Pseudomonas fluorescens* SS101 or its *cysH*-mutant.

	model term	df1	df2	F.ratio	p.value
Xca	Rhizobacteria	2	Inf	43.681	<.0001
	Genotype	1	Inf	7.058	0.0079
	Rhizobacteria:Genotype	2	Inf	17.234	<.0001
Xcc	Rhizobacteria	2	Inf	2.878	0.0562
	Genotype	1	Inf	6.523	0.0106
	Rhizobacteria:Genotype	2	Inf	2.253	0.105

Table S5. Level of three glucosinolates (GLSs: glucoiberin, glucoraphanin and glucobrassicin) in the shoot of the two Broccoli cultivars, Coronado and Malibu. Presented here are the average levels of these three GLSs in untreated plants (control) and their fold changes in plants treated with *Pf*SS101 or its *cysH*-mutant. *Pf*SS101: *Pseudomonas fluorescens* SS101, 20H12: *cysH*-mutant of *Pf*SS101.

Compound	GLS type	Average (n=4)		Fold change*			
		Control of Coronado	Control of Malibu	Coronado		Malibu	
				*Pf*SS101	20H12	*Pf*SS101	20H12
Glucobrassicin	Indole	44566275	32101930	5.09	1.89	3.80	1.97
Glucoiberin	Aliphatic	443556386	8324029	1.15	-1.12	1.45	1.30
Glucoraphanin	Aliphatic	659942362	575245511	1.22	-1.08	1.16	-1.03

* Fold changes were calculated relative to the untreated controls of each of the two Broccoli cultivars

Supplementary tables presented as excel files

Table S6. Annotation of Arabidopsis shoot metabolites that showed significant change in their abundance after root inoculation with rhizobacterium *Pf*SS101: *Pseudomonas fluorescens* SS101, or its mutant 20H12: *cysH*-mutant of *Pf*SS101. Fold changes were calculated by dividing the abundance of each metabolite to the non-treated control. Cluster numbers are associated with the cluster in **Figure 3B.**

Table S7. Annotation of Broccoli shoot metabolites that showed significant change in their abundance after root inoculation with rhizobacterium *Pf*SS101: *Pseudomonas fluorescens* SS101, or its mutant 20H12: *cysH*-mutant of *Pf*SS101. Fold changes were calculated by dividing the abundance of each metabolite to the non-treated control. Cluster numbers are associated with the cluster in **Figure 4B.**
